# Serum d-serine accumulation after proximal renal tubular damage involves neutral amino acid transporter Asc-1

**DOI:** 10.1038/s41598-019-53302-2

**Published:** 2019-11-13

**Authors:** Masataka Suzuki, Yusuke Gonda, Marina Yamada, Arno A. Vandebroek, Masashi Mita, Kenji Hamase, Masato Yasui, Jumpei Sasabe

**Affiliations:** 1 Keio University School of Medicine, Department of Pharmacology, Tokyo, 160-8582 Japan; 2 Nippon Sport Science University, Faculty of Medical Science, Kanagawa, 227-0033 Japan; 3 KAGAMI Lab, Shiseido Co., Ltd., 1-6-2 Higashi-shimbashi, Minato-ku, Tokyo 105-8310 Japan; 4 Kyushu University, Graduate School of Pharmaceutical Sciences, Fukuoka, 812-8582 Japan

**Keywords:** Kidney, Transporters, Biomarkers

## Abstract

Chiral separation has revealed enantio-specific changes in blood and urinary levels of amino acids in kidney diseases. Blood d-/l-serine ratio has been identified to have a correlation with creatinine-based kidney function. However, the mechanism of distinctive behavior in serine enantiomers is not well understood. This study was performed to investigate the role of renal tubules in derangement of serine enantiomers using a mouse model of cisplatin-induced tubular injury. Cisplatin treatment resulted in tubular damage histologically restricted to the proximal tubules and showed a significant increase of serum d-/l-serine ratio with positive correlations to serum creatinine and blood urine nitrogen (BUN). The increased d-/l-serine ratio did not associate with activity of a d-serine degrading enzyme, d-amino acid oxidase, in the kidney. Screening transcriptions of neutral amino acid transporters revealed that Asc-1, found in renal tubules and collecting ducts, was significantly increased after cisplatin-treatment, which correlates with serum d-serine increase. *In vitro* study using a kidney cell line showed that Asc-1 is induced by cisplatin and mediated influx of d-serine preferably to l-serine. Collectively, these results suggest that cisplatin-induced damage of proximal tubules accompanies Asc-1 induction in tubules and collecting ducts and leads to serum d-serine accumulation.

## Introduction

Mammals utilize l-amino acids in most biological processes^1^. Blood amino acids consist predominantly of their L-forms^2–5^, while urinary amino acids include fair amount of d-forms^4,6^. The kidney appears to play a pivotal role in maintaining the homochirality of blood amino acids through stereoselective reabsorption of l-amino acids. d-Serine, an enantiomer of l-serine, is a major exception in such biological homochirality of amino acids in mammals^1^. d-Serine is converted from l-serine by a mammalian pyridoxal-5′-phosphate- (PLP-) dependent enzyme^7^ and comprises one fourth of total serine in the central nervous system^8^. d-Serine modulates glutamatergic neurotransmission in the central nervous system although knowledge of its functional roles in the periphery is limited. Blood d-serine is filtered in the renal glomeruli, partially reabsorbed in the proximal tubules, and the remainder is excreted in the urine. Alanine-serine-cystein-1 (Asc-1) transporter in neurons and ASCT1 transporter in astrocytes play a primary role for d-serine transport in the central nervous system^9–12^, whereas d-serine transport in the proximal tubules remains largely unknown. The reabsorbed d-serine through unknown mechanism is known to be degraded by d-amino acid oxidase (DAO) expressed at highest levels in the kidney proximal tubules in mammals^13^. Interestingly, recent studies on both rodents and humans show that blood d-serine levels are increased with kidney dysfunction and that d-serine can serve as an emerging biomarker for kidney dysfunction because of robust positive correlation to serum creatinine in ischemic acute kidney injury (AKI) or chronic kidney diseases (CKD)^4,5,14^. On the contrary, blood l-serine levels are significantly reduced with kidney dysfunction most probably due to reduced tubular reabsorption^4,5^. Given that part of filtered d-serine as well as l-serine is uptaken by the kidney tubules via neutral amino acid transporters, it is unclear how differently serine enantiomers are reabsorbed along the nephrons and why the dynamics of serine enantiomers differ under kidney dysfunction in the opposite ways.

Cisplatin is a widely used and successful drug for the treatment of various tumors such as ovarian, testicular, head and neck, lung, bladder and other solid tumors^15^. However, it often causes clinical problems: ototoxicity, neurotoxicity, and nephrotoxicity. Nephrotoxicity by cisplatin is described as damages to renal proximal tubules from basolateral but not luminal side^16^. Cisplatin, transported by copper transporter 1 and organic cation transporter 2 located on the basolateral membrane of renal proximal tubules^17^, forms DNA adducts, generates oxidative stress, stimulates apoptotic signals such as MAPK, releases inflammatory cytokines, and finally induces tubular cell apoptosis^18^.

To understand the mechanism underlying blood d-serine, but not l-serine, accumulation in kidney dysfunction, we developed an animal model with selective proximal tubular damage induced by moderate dose of cisplatin. We demonstrate whether selective proximal tubular damage accumulates blood d-serine stereoselectively, and if yes, what kind of d-serine modulating factors, including DAO activity and d-serine transport, is involved in blood d-serine accumulation.

## Results

### Evaluation of renal damage in cisplatin-treated mice

**Figure 1:**
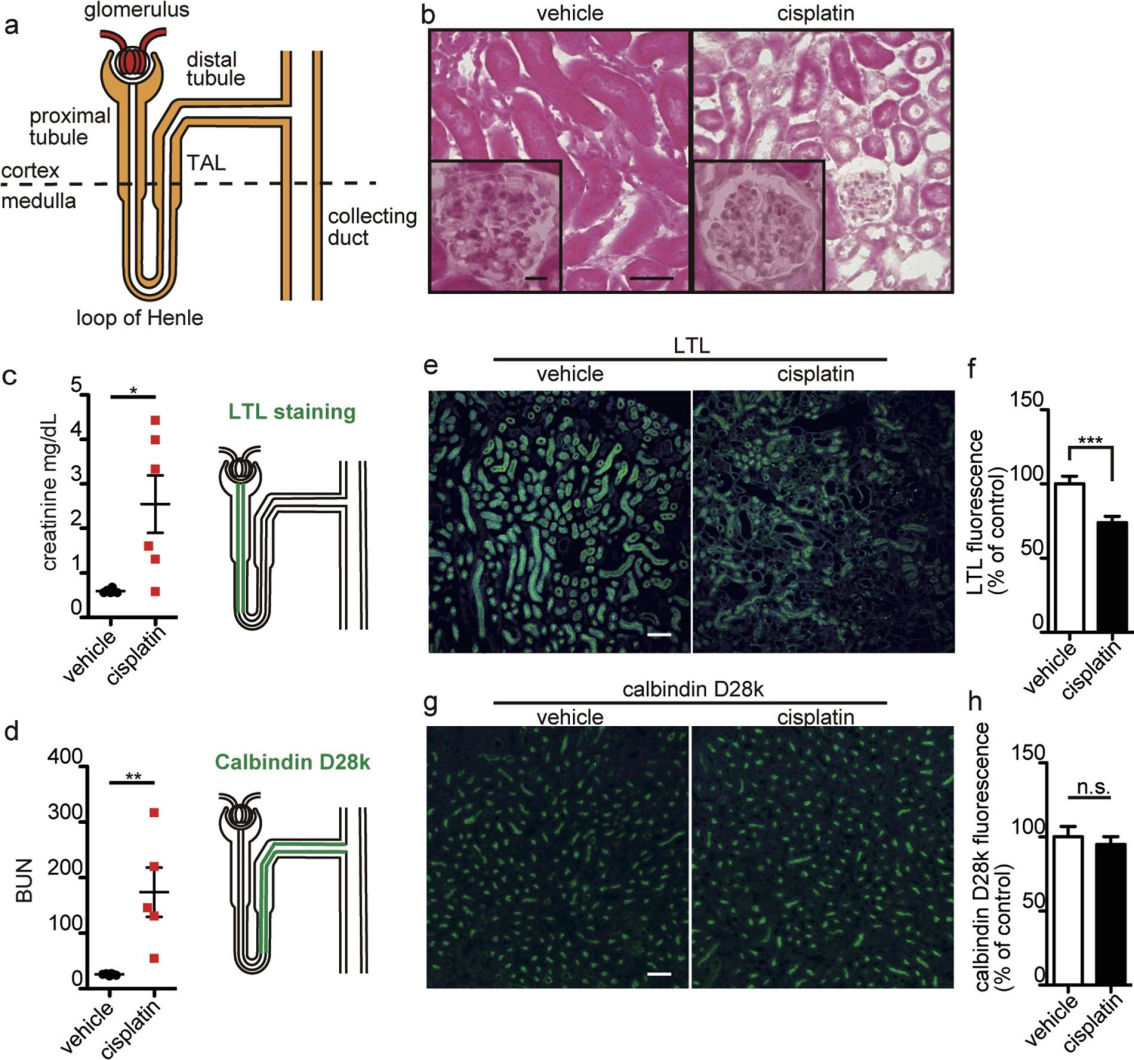
Disrupted renal proximal tubules in cisplatin-treated mouse. (**a**) The structure of a nephron is illustrated. (**c**,**d**) Serum creatinine (n = 6 each) (**c**) and BUN (vehicle, n = 6; cisplatin, n = 5) (d) in mice at 72 hours after injection with vehicle or cisplatin are shown. Error bars, mean ± s.e.m. Mann-Whitney U test, *P < 0.05, **P < 0.01, ***P < 0.001 (**c**,**d**). ‘n.s.’, not significant. Sections of kidneys from vehicle- or cisplatin-treated mice were stained with H&E (**b**), or labeled with fluorescent-LTL (green, **e**) or an antibody to calbindin-D28k (green, **g**). Nuclei are stained with DAPI (blue, **e**,**g**). Images are from renal cortices (**b**,**e**) and cortex/medulla (**g**). Fluorescence intensities of LTL- (vehicle, 25 areas in 5 mice; cisplatin, 30 areas in 6 mice) or calbindin-D-28k-positive tubules (vehicle, 26 areas in 3 mice; cisplatin, 26 areas in 3 mice) were quantified using densitometry (**f**,**h**). Scale bars, 50 µm. Inlets in (**b**) are enlarged glomeruli.

Given the d-serine dysregulation found in AKI, we tested if cisplatin-induced nephrotoxicity disturbs d-serine homeostasis in mice. First, we evaluated renal pathology in the mice injected with cisplatin at the lowest (20 mg/kg) dose that causes nephrotoxicity (Fig. 1a,b)^19,20^. Cisplatin-treated group produced a significant increase in serum creatinine and blood urea nitrogen (BUN) levels compared to vehicle-treated controls (Fig. 1c,d). Hematoxylin and eosin (H&E) staining showed the increase in damaged tubular cells with no apparent morphological changes of glomeruli in the renal cortex of cisplatin-treated mice. Fluorescence-conjugated lotus tetragonolobus lectin (LTL) was used to identify proximal tubules^21,22^, and cisplatin-injection significantly reduced LTL-positive tubules in the renal cortex (Fig. 1e,f). Since LTL binds carbohydrate of proximal tubules especially on the apical side, the reduced LTL-reactivity reflects cisplatin-induced histological changes including loss of the apical brush border on proximal tubules^23^. Cisplatin-treatment did not affect tubules labeled with calbindin-D28k, exclusively localized in the distal tubules and in the proximal part of the collecting ducts (Fig. 1g,h)^24,25^. Therefore, the cisplatin-treated mice used in this study showed renal injury histologically restricted in the proximal tubules.

### Altered d-serine level in the serum and urine by cisplatin treatment

**Figure 2:**
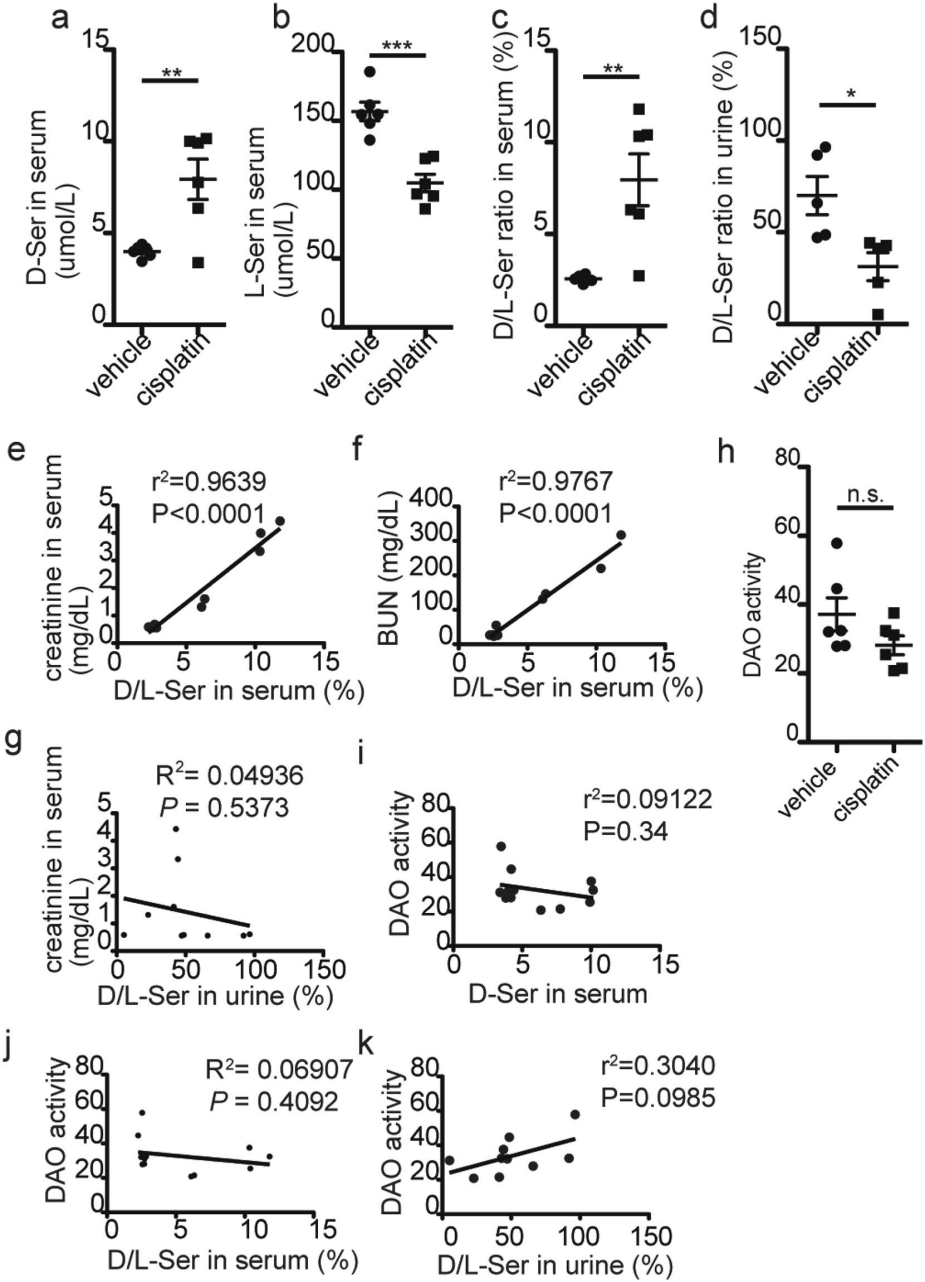
Impaired homeostasis of serine enantiomers in cisplatin-treated mice. Serum and urine d- and L-serine levels or d-/l-serine ratios at 72 hours after the injection of cisplatin were measured using the 2D-HPLC system. (**a**–**d**) Serum d-serine (**a**) and l-serine (**b**), serum d-/l-serine ratio (**c**), and urine d-/l-serine ratio (**d**) in vehicle- and cisplatin-treated mice are shown (serum, n = 6 each; urine, n = 5 each). Error bars, mean ± s.e.m. Mann-Whitney U test, *P < 0.05, **P < 0.01, ***P < 0.001. (**e**–**g**) Correlation of d-/l-serine ratio in the serum (**e**,**f**) or urine (**g**) with serum creatinine (**e**,**g**) and BUN (**f**) were analyzed (Friedman’s test). (**h**) The plots indicate DAO activity in whole kidney of vehicle- or cisplatin-treated mice (n = 6 each). Error bars, mean ± s.e.m. Student’s t-test. ‘n.s.’, not significant. (**i**–**k**) Correlations of DAO activity to amount of d-serine in the serum (**i**) and d-/l-serine ratio in the serum (**j**) or urine (**k**) are shown. Pearson’s correlation is shown on figure respectively.

For quantification of serine enantiomers, we used two-dimensional high performance liquid chromatography (2D-HPLC) with sensitive and specific separation of d- and l-serine (Supplementary Fig. S1). The cisplatin-treated group showed 2.0-fold increase in serum d-serine (vehicle: 4. 01 ± 0.13 µmol/L, cisplatin: 7.93 ± 1.10 µmol/L)(*P* = 0.0054, Fig. 2a), 1.5-fold decrease in l-serine (vehicle: 156.8 ± 6.76 µmol/L, cisplatin: 104.9 ± 6.33 µmol/L)(*P* = 0.0002, Fig. 2b), and thereby 3.1-fold increase in d-/l-serine ratio (vehicle: 2.57 ± 0.08%, cisplatin: 7.95 ± 1.41%)(*P* = 0.0034, Fig. 2c) compared to vehicle controls. In contrast, d-/l-serine ratio in the urine was reduced by 2.2-fold after cisplatin-treatment (vehicle: 70.05 ± 10.43%, cisplatin: 31.34 ± 7.62%) (*P* = 0.0172, Fig. 2d). Strikingly, there were very strong positive correlations between serum d-/l-serine ratio and both serum creatinine (R^2^ = 0.9639, Fig. 2e) and BUN (R^2^ = 0.9767, Fig. 2f) but no association between urine d-/l-serine ratio and serum creatinine (R^2^ = 0.04936, Fig. 2g), suggesting that serum d-/l-serine ratio is an excellent indicator for renal function and the variation of serum d-serine-levels observed in the cisplatin-treated group reflects the individual variability in the renal damage.

DAO is highly expressed in renal proximal tubules and renal ischemic reperfusion injury was reported to reduce renal DAO activity by 30–45% in rodents^4^. Unexpectedly, cisplatin-induced acute kidney injury did not significantly alter renal DAO activity (*P* = 0.1355, Fig. 2h), which suggested renal DAO activity was preserved even with relatively severe proximal tubular damage. Renal DAO activity was not significantly associated with serum d-serine level (Fig. 2i) or serum/urine d-/l-serine ratios (Fig. 2j,k), supporting the view that alterations of d-/l-serine ratio in the serum and urine were not caused by altered DAO activity.

### Correlation between transcriptional levels of amino-acid transporters and serum d-serine level in cisplatin-treated animals

**Figure 3:**
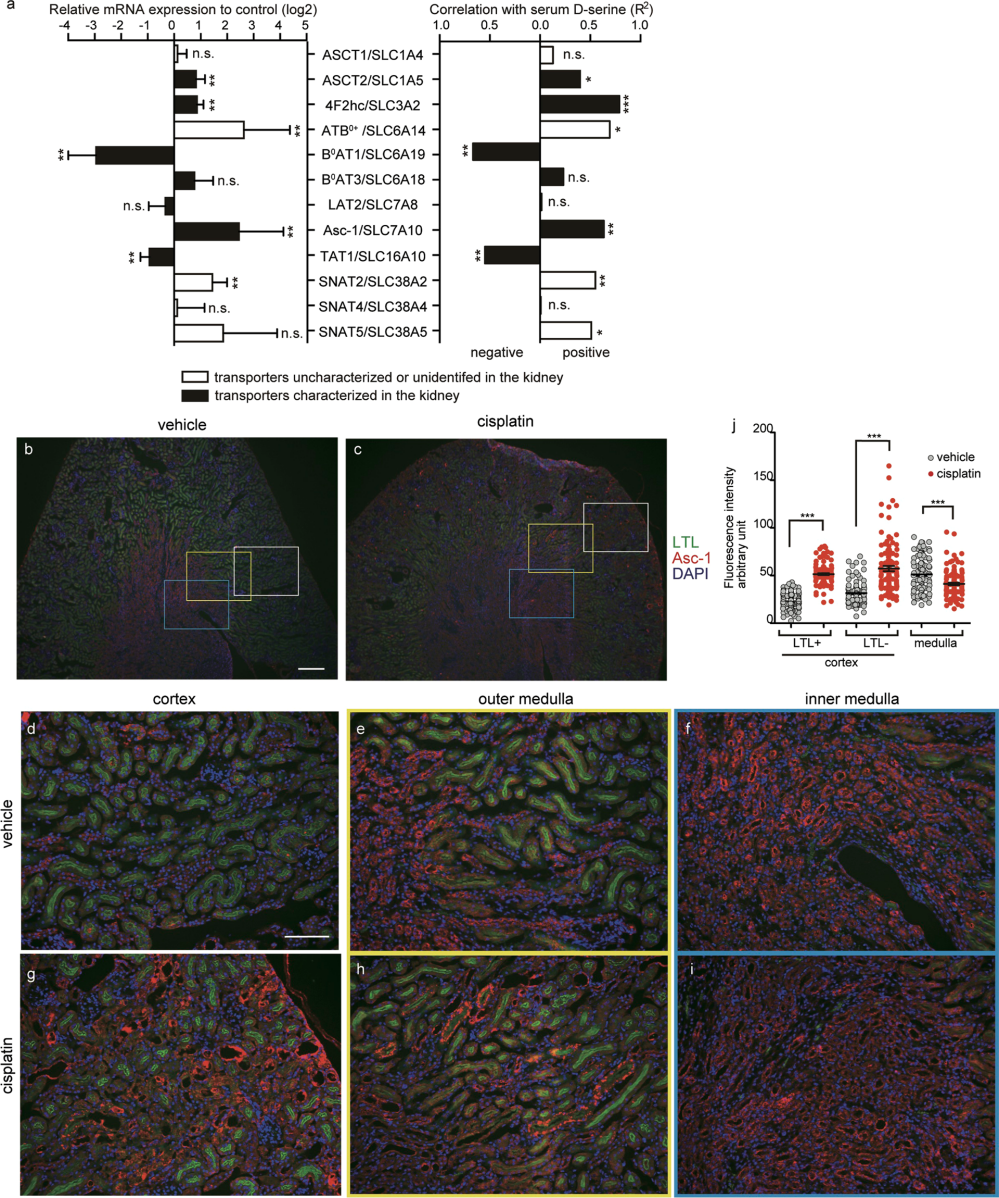
Expressions of d-serine transporters in the cisplatin treated kidney. (**a**, left panel) mRNA expression of d- and l-serine transporters are indicated. Error bars, geometric mean with 95% c.i. Mann-Whitney U test. (**a**, right panel) R square value of correlation of serum d-serine to mRNA levels of d-/l-serine transporters in the whole kidney is shown. *P < 0.05, **P < 0.01, ***P < 0.001. ‘n.s.’ shows ‘not significant’. (**b**–**i**) LTL (green), Asc-1 (red), and DAPI (blue) were stained using kidney slices from vehicle (**b**,**d**–**f**)- and cisplatin (**c**,**g**–**i**)-treated mice. Representative images of renal cortex (**d**,**g**), outer medulla (**e,h**), and inner medulla (**f**,**i**) are shown. Scale bar indicates 300 μm (**b**,**c**) or 100 μm (**d**–**i**). (**j**) Fluorescence intensities of Asc-1 labelling in 20 renal tubules in either renal cortex or medulla of the kidney slices from each mice treated with vehicle or cisplatin were analyzed (vehicle, n = 5; cisplatin, n = 6). Error bars, mean ± s.e.m. Mann-Whitney U test, ***P < 0.001.

Renal tubules express a variety of amino-acid transporters, including those for neutral amino acids^26^. We next analyzed renal mRNA expression patterns of diverse neutral amino acid transporters to study association with serum d-serine level altered by cisplatin treatment. Among the transporters studied, proximal tubules are known to express ASCT2^27,28^, B^0^AT1, and B^0^AT3 on the apical membrane^29^, while LAT2^30^ and TAT1^31^ are localized on the basolateral side. In contrast, Asc-1 is reported exclusively in the distal tubules and collecting duct^32^. Major expressors of the other transporters include the nervous systems (ASCT1^12^, SNAT5^33^), intestine (ATB^0+^)^34^, and liver (SNAT2^35^, SNAT4^36^). Accessory protein 4F2hc for SLC7 family, such as LAT2 and Asc-1, is found ubiquitously. Cisplatin-treatment on mice significantly reduced the transcription of B°AT1 and TAT1, while it elevated transcription of ASCT2, 4F2hc, and Asc-1 (Fig. 3a *left*). On the other hand, transcription of non-renal transporters ATB^0+^ and SNAT2 was increased in the kidney of cisplatin-treated animals (Fig. 3a *left*), although their physiological or pathological roles in the kidney have not been identified yet. Serum d-serine levels were positively correlated with transcriptions of ASCT2, 4F2hc, ATB^0+^, Asc-1, SNAT2, and SNAT5 and negatively with those of B^0^AT1 and TAT1 (Fig. 3a *right*, Supplementary Fig. S2). Therefore, among renal transporters for neutral amino acids^26^, ASCT2, 4F2hc, and Asc-1 showed elevated transcription with positive correlation with serum d-serine.

### Characteristic of serine enantiomers transport by Asc-1

Asc-1 has been regarded as the primary transporter for d-serine in the central nervous system^9–11^. Expression of Asc-1 mRNA is localized to Henle’s loop, distal tubules, and collecting ducts^32^, but d-serine transport by Asc-1 in the kidney has remained uncharacterized. A database (JASPAR) of transcription-factor binding profiles^37^ shows that the mouse Asc-1 promoter region includes potential binding sites for stress-activated or inflammatory cytokine-activated transcription factors, such as FOS::JUN, NF-kB, and STAT3 (Table S1). As cisplatin-treatment is known to trigger oxidative stress, activate MAPKs^38^, and release inflammatory cytokines in the renal proximal tubules^39,40^, this information support the idea that enhanced Asc-1 transcription is associated with cisplatin-induced responses. To confirm expressional change of Asc-1 in the kidney of cisplatin-treated animals, we performed histological analysis to understand tissue distribution of Asc-1. We raised a polyclonal antibody against an N-terminal epitope of mouse Asc-1. Immunoprecipitation and immunocytochemistry with the Asc-1 antibody on HEK293 cells overexpressed with FLAG-tagged Asc-1 suggested that the antibody recognizes the steric structure of Asc-1 (Supplementary Fig. S3). Asc-1 was not highly expressed in the cortex or medulla of kidney in control animals treated with vehicle, while cisplatin treated animals with mild renal damage (serum creatinine < 3 mg/dL) showed a significant increase in especially apical expression in both LTL-positive and negative tubules in the cortex but not in the medulla (Fig. 3b–j). Mice with severe renal damage (serum creatinine > 3 mg/dL), which did not exhibit many intact proximal tubules in the renal cortex, showed a strong increase of Asc-1 expression in non-LTL-positive structure (Supplementary Fig. S4). These results suggest that Asc-1 can work as a supplementary amino-acid transporter under pathologic conditions where proximal tubules are damaged.

**Figure 4:**
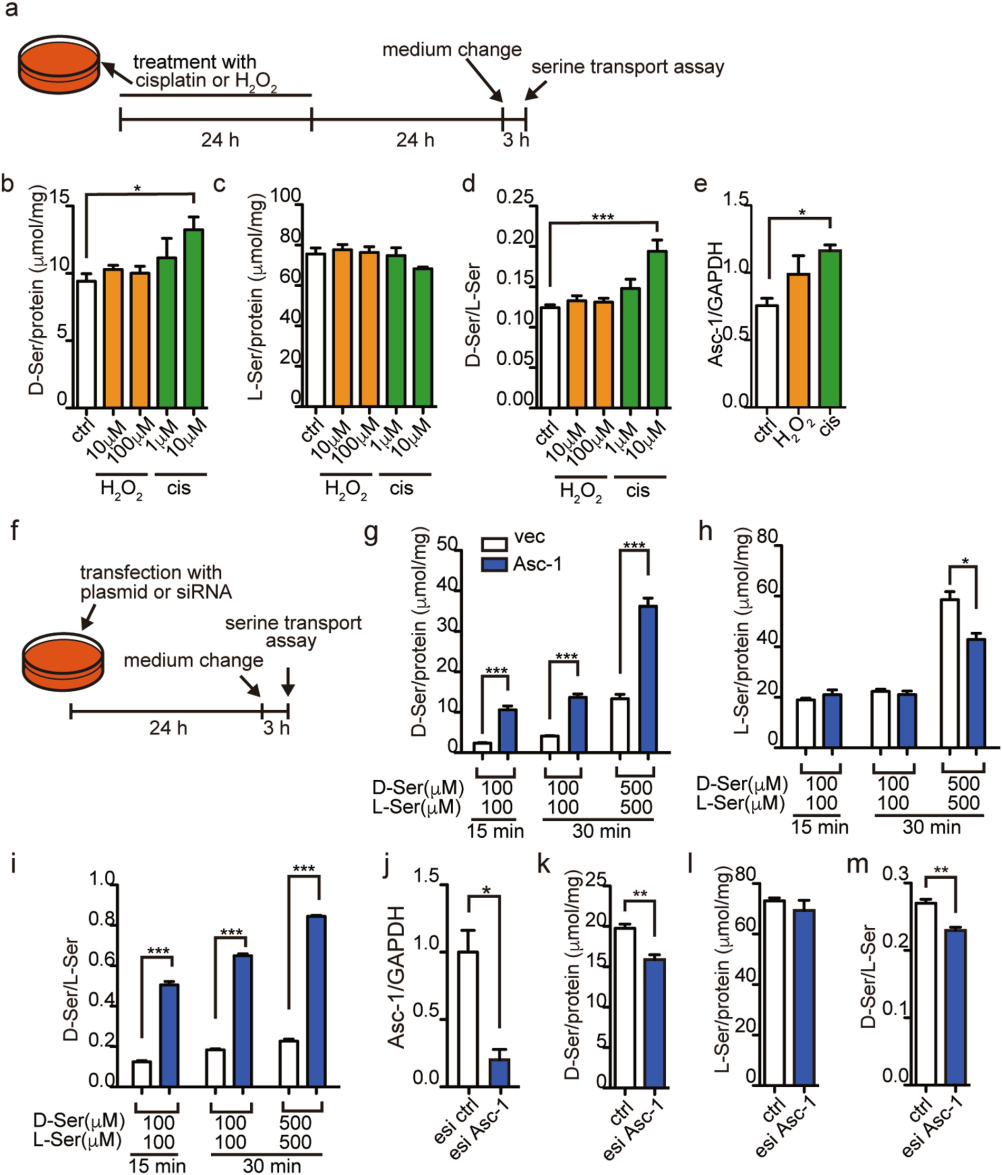
Involvement of Asc-1 in d-serine transport under cisplatin treatment. Inward transport of d- and l-serine in the HEK293 cells was measured after treatment with cisplatin or H_2_O_2_ (**a**–**d**), or after overexpression or knockdown of Asc-1 (**f**–**i**,**k**–**m**). (**a**) Schematic shows details of treatments for (**b**–**d**). (**b**–**d**) Transported intracellular d-serine (**b**), l-serine (**c**), and d-/l-serine ratio (**d**) in HEK293 cells treated with cisplatin or H_2_O_2_ were quantified using 2D-HPLC. The amounts of amino acids were standardized with protein quantify in the cells. (**e**) Expression of mRNA for Asc-1 after treatment with 10 μM cisplatin or H_2_O_2_ for 15 h was evaluated with qPCR and standardized with mRNA levels of GAPDH. (**f**) Schematic shows details of treatments for (**g**–**I**,**k**–**m**). (**g**–**i**,**k**–**m**) Transported intracellular d-serine (**g**,**k**), l-serine (**h**,**l**), and d-/l-serine ratio (**i**,**m**) in HEK293 cells overexpressed with Asc-1 (**g**–**i**) or knocked-down of Asc-1 (**k**–**m**) were quantified using 2D-HPLC. The amounts of amino acids were standardized with protein quantity in the cells. (**j**) Expression of mRNA for Asc-1 at 24 h after transfection with esiAsc-1 or its control was evaluated with qPCR and standardized with mRNA levels of GAPDH. ‘cis’, cisplatin. ‘ctrl’, control. Biological replicates, n = 4 for each condition. Error bars, mean ± s.e.m. Student’s t-test. *P < 0.05, **P < 0.01, ***P < 0.001.

We further tested if cisplatin can increase transport of serine enantiomers with induction of Asc-1 *in vitro* using a modified 2D-HPLC system (Fig. 4a, Supplementary Fig. S5a–d). To evaluate uptake of d- and l-serine, we first washed out intracellular l-serine, which was accumulated in the HEK293 cells cultured in growing medium, by replacement with serum free essential medium (Supplementary Fig. S5e), and then treated the cells with a racemic mixture of d- and l-serine. Stimulation with cisplatin for 24 hours on HEK293 cells increased transport of d-serine (Fig. 4b) but not of l-serine (Fig. 4c) and resulted in elevation of d-/l-serine ratio within the cells (Fig. 4d), while H_2_O_2_ did not affect both serine enantiomers (Fig. 4b–d). At the same time, cisplatin treatment elevated mRNA expression of Asc-1 (Fig. 4e), supporting the association of Asc-1 with increased transport of d-serine. Furthermore, overexpression of Asc-1 in HEK293 cells (Fig. 4f) caused a significant accumulation of intracellular d-serine in a dose dependent manner over time (Fig. 4g), while L-serine within the cells overexpressing Asc-1 was lower than that in vector-transfected cells (Fig. 4h). Therefore, overexpression of Asc-1 resulted in increased intracellular d-/l-serine ratio (Fig. 4i). On the other hand, knockdown of Asc-1 using endoribonuclease-prepared siRNA (esiRNA) in HEK293 cells (Fig. 4j) significantly reduced uptake of d-serine but not l-serine and resulted in decreased intracellular d-/l-serine ratio (Fig. 4k–m). These results suggest that Asc-1 mediates inward transports of d-serine preferably to l-serine. Collectively, our findings support the idea that induction of Asc-1 by cisplatin accelerates d-serine transport from urinary tract to the kidney tubules.

## Discussion

We found an increase of serum d-serine level with a positive correlation to serum creatinine in a cisplatin-induced AKI mouse model. Cisplatin-treatment induces expression of some neutral amino acid transporters, including Asc-1, in renal tubules and collecting ducts. *In vitro*, cisplatin increases cellular influx of d-serine but not that of l-serine with an induction of Asc-1 in a kidney cell line.

Damage to proximal kidney tubules induced by cisplatin results in an elevation of d-/l-serine ratio in the serum, while at the same time a reduction of the ratio in the urine (Fig. 2c,d). As proximal tubules are known to express several neutral amino acid transporters^26^, including B^0^AT1, B^0^AT3, and LAT2, disturbed reabsorption after tubular damage can directly lead to decreased levels of serum l-serine (Fig. 2b), increased levels of urinary l-serine, and a resultant alteration of d-/l-serine ratio in the serum and urine. Indeed, expression of a neutral amino acid transporter B^0^AT1 that has high affinity for l-serine is significantly reduced after cisplatin treatment (Fig. 3a). Mutations in the B^0^AT1 gene cause Hartnup disease, which triggers abundant urinary excretion of neutral amino acids^41^, suggesting downregulation of B^0^AT1 may play a central role in such alterations of serum and urinary l-serine levels. Together with the previous reports that reduced serum l-serine and increased urinary excretion of l-serine is commonly found in mice after kidney ischemic reperfusion injury (IRI)^4^ or in chronic kidney diseases^5^, downregulation of B^0^AT1 may play a critical role in the regulation of l-serine in the kidney diseases.

In contrast to l-serine, d-serine is accumulated in the serum (Fig. 2a), suggesting the presence of other mechanisms than down-regulation of B^0^AT1 in the cisplatin-induced tubular damage. DAO, which is expressed at highest levels in the renal proximal tubules in mammals, degrades multiple neutral and basic d-amino acids including d-serine. In serum from DAO-deficient mice, d-serine is significantly accumulated^42^, and therefore, tubular DAO is assumed to degrade reabsorbed d-serine from the luminal side to avoid transition of the amino acid into basolateral side. In fact, in DAO deficient mice, renal IRI attenuated time-dependent d-serine accumulation in the serum^4^, supporting the idea that serum d-serine increase, at least in part, involves reduced DAO activity in the IRI-induced acute kidney injury. Unexpectedly, however, in the present study, we show that cisplatin treatment does not significantly reduce kidney DAO activity, indicating that d-serine accumulation in the kidney diseases occurs also through a mechanism independent of DAO. d-Serine is known to have relatively high affinity to neutral amino acid transporters such as Asc-1, ASCT2, and ATB^0+^. In the central nervous system, knockout of Asc-1 but not ASCT2 increases d-serine levels in the synaptic clefts^9^, showing that Asc-1 is a primary d-serine transporter. ATB^0+^ expressed in the colon is a Na^+^ and Cl^−^- coupled transporter for l-enantiomers of neutral and cationic amino acids and is also capable of mediating d-serine transport with high-affinity^43^. The kidney expresses ASCT2 in the proximal tubules, and Asc-1 in the distal tubules and collecting ducts, but expression of ATB^0+^ in the kidney remains uncertain. Although none of them are known to work as major neutral amino acid transporters in the kidney, cisplatin-treatment induces mRNA expression of Asc-1, ASCT2, and ATB^0+^ with a positive correlation to serum d-serine levels (Fig. 3a). Since Asc-1 plays a major role in d-serine transport in the central nervous system, in this study, we focused on Asc-1 and found that Asc-1 is inducible in the presence of cisplatin (Figs 3b–j and 4e) and contributes to cellular influx of d-serine (Fig. 4f–m). Although, to the best of our knowledge, inflammatory induction of Asc-1 in the central nervous system has not been reported, Asc-1 can potentially be induced under inflammatory stimuli since mouse Asc-1 promoter region includes potential binding sites for stress-activated or inflammatory cytokine-activated transcription factors (Table S1). Considering that kidney induction of Asc-1 by cisplatin is not restricted in proximal tubules but also found in distal tubules and collecting ducts (Fig. 3b–j and Supplementary Fig. S4), we speculate that proinflammatory nature by cisplatin could influence inflammatory cells of the immune system such as T cells, macrophages, neutrophils, and mast cells to infiltrate the kidney tissue and eventually induce Asc-1^44^. From functional aspect, induction of Asc-1 may compensate for impaired reabsorption of neutral amino acids in the damaged proximal tubules in the cisplatin-treated animals (Fig. 1). Therefore, our findings raise a possibility that Asc-1, capable of high-affinity transport of d-serine, induced in the distal tubules or collecting ducts, where DAO is not expressed, may trigger serum d-serine accumulation. Induction of Asc-1 may also influence serum L-serine reduction (Fig. 2b) or glycine level since Asc-1 mediates the bidirectional transport of d-serine coupled with the counter-transport of small neutral amino acids, referred to as the exchange mode^45,46^. Furthermore, we also investigated *in vitro* consequence of cisplatin treatment on ASCT2, which has much lower affinity for d-serine (Km = 1 mM) compared to Asc-1 (Km = 20–52 µM). Cisplatin also induced ASCT2 *in vitro* and *in vivo*, and ASCT2 transported d-serine in a similar manner with Asc-1 in the kidney cell line (Supplementary Fig. S6). These results do not exclude the possibility that ASCT2 may also compensate for impaired tubular amino acid transport and contribute in part to serum d-serine accumulation in the cisplatin induced tubular damage. However, since cisplatin inhibits reabsorption of sodium at proximal tubules^47,48^, Asc-1, a sodium-independent antiporter, could uptake more efficiently than ASCT2, a sodium-dependent antiporter, in the cisplatin treated animals.

Thus, in the present study, we have shown a potential mechanism underlying serum d-serine accumulation in the acute kidney tubular damage. Although transports of d-amino acids still remain largely unclear in the kidney and intestine, recent reports that commensal microbes produce diverse d-amino acids^49–51^ warrant further studies of d-amino acid transport in mammals.

## Methods

### Animals

All animal experiments were approved by the institutional Animal Experiment Committee and conducted in accordance with Institutional Guidelines on Animal Experimentation at Keio University. C57BL/6Jjcl male mice at 6 weeks old were purchased from CLEA Japan (Tokyo, Japan). At three days after intraperitoneal injection of cisplatin (20 mg/kg), mice were euthanized by inhalation of isoflurane. The blood was collected from inferior vena cava in BD microtainer tube with serum separator and centrifuged at 700 × g for 10 min. Urine was collected from the bladder by puncture aspiration. The serum and urine were stored at −80 °C until use. Serum creatinine and blood urine nitrogen (BUN) was quantified using the Fuji DRI-CHEM 4000 system (Fujifilm, Tokyo, Japan).

### Antibodies

A polyclonal antibody against mouse Asc-1 was custom produced by GenScript (Piscataway, NJ, USA). In short, peptide of an N-terminal epitope (MRRDSDMASHIQQPC) of mouse Asc-1 was synthesized and immunized in two rabbits. Polyclonal antiserum was affinity-purified using the peptide antigen and had ELISA titers of 1:128,000 (validated with immunoprecipitation and immunocytochemistry in Supplementary Fig. S1). Rabbit polyclonal antibody to calbindin D28K was obtained from Sigma Aldrich (St Louis, MO, USA).

### Cloning, transfection, and immunoprecipitation of Asc-1

A mouse *Asc-1* cDNA (NM_017394) was PCR-amplified from cDNA library isolated from the mouse cerebral cortex with a sense primer (ATGAGGCGGGACAGCGAC) and an antisense primer (TCATTGTGTCTTCAAGGGCTTG). cDNA for *Asc-1* “was subcloned into the pFLAG-CMV5a vector (Sigma Aldrich) using an In-Fusion HD cloning kit (Takara-clontech, Shiga, Japan) (a sense primer, ATCAGTCGACGGATCCACCATGAGGCGGGACAGCGAC; an antisense primer, AATCGGTACCGGATCCTCATTGTGTCTTCAAGGGCTTG). Sequence was confirmed using primers (AATGTCGTAATAACCCCGCCCCGTTGACGC, CTATGTGCTTCAGCCTGTCT, and GAGGGATCAATGGCTACCTG) (Table S2). HEK293 cells were cultured in 10% FBS D-MEM for 1 day and transfected with pFLAG Asc-1 using Lipofectamine 2000 (ThermoFisher Scientific, MA, USA) according to the manufacturer’s protocol. At 24 h after transfection, cells were lysed in an immunoprecipitation (IP) buffer [50 mM HEPES-NAOH pH 7.4, 150 mM NaCl, 1 mM EDTA, 0.1% TritonX-100, a protease inhibitor cocktail (cOmplete, Merck, Darmstadt, Germany)], sonicated for 20 sec on ice, and centrifuged at 12,100 × g for 5 min. Each lysate was divided into two samples and mixed with rabbit normal IgG or a rabbit polyclonal antibody to Asc-1 that are conjugated with protein G sepharose beads (GE healthcare) overnight. Then the beads were washed in the IP buffer four times and mixed with a sample buffer [100 mM Tris-HCl pH 6.8, 5% (w/vol) SDS, 25% (vol/vol) glycerol]. Samples were applied to SDS-PAGE without denaturation and transferred to PVDF membrane. After being blocked in 5% skim milk-TBST at room temperature for 1 h, the membrane was incubated with a mouse monoclonal antibody to FLAG-tag (Sigma Aldrich, MO, USA).

### Histology

Euthanized mice were perfused with ice-cold PBS (pH7.4). Tissue was dissected, fixed in 4% paraformaldehyde PBS for 2 hours, and dehydrated in 20% sucrose PBS at 4 °C overnight. The tissue was embedded in a solution [OCT compound: 20% sucrose PBS = 2:1] and sliced to 10 µm-thickness on a cryostat at −19 °C and stored at −80 °C until use.

For H&E staining, tissue sections were washed in PBS, stained with hematoxylin and eosin, dehydrated, cleared, and mounted with Entellan new (Merck, Darmstadt, Germany).

For immunofluorescent staining, tissue sections were washed in PBS. After removal of endogenous peroxidase activity with incubation in 0.3% H_2_O_2_ PBS for 10 min, the sections were blocked in 5% goat serum PBS for 1 hour, immersed in rabbit antibodies to calbindin D28K (Sigma Aldrich) or Asc-1 at 4 °C overnight, incubated in a biotinylated goat antibody to rabbit IgG for 30 min, and then labeled with streptavidin-HRP in PBS for 30 min. After being washed, the slides were incubated in Cy3-conjugated tyramid (1:200, Perkin-Elmer, Waltham, MA, USA) for 10 min, rinsed in PBS three times, and coverslipped with Prolong Gold Antifade Reagent with DAPI (ThermoFisher scientific, Waltham, MA, USA).

Sections were visualized using microscope BZ-9000 (Keyence, Osaka, Japan). Each section being compared was imaged under identical conditions. Fluorescence intensity in each section was measured using Fiji software (http://fiji.sc/). The intensities were measured in each tubule and standardized by area using Fiji. Each tubular fluorescence signal was subtracted from the background signal in an identical manner for each section being compared.

### Immunocytochemistry

HEK293 cells were cultured on chamber slides coated with 0.01% collagen type II and transfected with pFLAG-Asc-1 using lipofectamine 2000 (ThermoFisher Scientific, MA, USA) according to the manufacturer’s protocol. At 24 hours after transfection, cells were washed in PBS, fixed in 4% paraformaldehyde PBS at 4 °C for 2 h, and immersed in a blocking buffer [5% normal goat serum, 0.3% TritonX-100 PBS] at 4 °C for 1 h. Then, cells were immunolabeled with a mouse monoclonal antibody to FLAG (1:100, Sigma Aldrich, MO, USA) and a rabbit polyclonal antibody to Asc-1 (1:100) in blocking buffer at 4 °C overnight. The cells were incubated with an FITC-conjugated goat antibody to mouse IgG and a Texas red-conjugated goat antibody to rabbit IgG (both 1:200, Jackson ImmunoResearch Laboratories, PA, USA).

### Quantification of serine enantiomers

d- and l-Serine in the serum and urine was quantified using a two-dimensional high performance liquid chromatography (2D-HPLC) system (NANOSPACE SI-2 series, Shiseido, Tokyo, Japan) as previously described^42^. Briefly, the serum and urine were deproteinized with methanol. The amino acids in the liquid layer were derivatized with 4-fluoro-7-nitro-2,1,3-benzoxadiazole (NBD-F). For quantification of d- and l- serine in plasma, NBD-amino acids were separated in two tandem-connected columns (a capillary-monolithic ODS column & a narrowbore-enantioselective column, provided from Shiseido), and detected at 530 nm with excitation at 470 nm. Supplementary Fig. S1 shows representative chromatograms of amino acid standards (Supplementary Fig. S1a,b) and amino acids in plasma (Supplementary Fig. S1c,d) in the first dimensional separation (1D: Supplementary Fig. S1a,c) and in the second dimensional one (2D: Supplementary Fig. S1b,d). For *in vitro* transport analysis, NBD-amino acids were separated in octadecylsilyl (ODS) column (KSAARP, 1.0 mm inner diameter (ID) × 250 mm) (designed by Kyushu University and Shiseido) for 1D separation and a pirkle-type enantioselective column (KSAACSP-001S, 1.5 mm ID × 250 mm) for 2D separation (designed by Kyushu University and Shiseido). Supplementary Fig. S5a–e show the chromatograms of amino acid standards (Supplementary Fig. S5a,b) and amino acids in cell lysate (Supplementary Fig. S5c,d) in the 1D separation (Supplementary Fig. S5a,c) and in the 2D (Supplementary Fig. S5b,d). Standard D- and L-serine were obtained from Wako (Osaka, Japan).

### DAO activity assay

DAO activity in the kidney was measured as described in previous reports^52^. Kidney was homogenized in 7 mM pyrophosphate buffer (pH 8.3) and centrifuged at 5,500 × g for 10 min. Fifty microliter of the supernatant was mixed with 150 μL of 700 IU/mL catalase, 150 μL of 1 mM d-alanine, 100 μL of 0.1 mM Flavin adenine dinucleotide and 100 μL of 70% methanol. After incubation at 37 °C for 1 h, 500 μL of 10% trichloroacetic acid was mixed and centrifuged at 24,500 × g for 5 min. The supernatant was mixed with same volumes of 5 M KOH and 0.5% 4-amino-3-hydrazino-5-mercapto-1,2,4-triazole, incubated at room temperature for 15 min and mixed with one-third volume of 0.75% KIO4 in 0.2 M KOH. Absorbance at 550 nm was measured, and DAO activity was calculated as described by Watanabe *et al*.^53^.

### Gene expression analysis

RNA was extracted from dissected kidneys or cultured cells using the RNAiso Plus reagent (Takara Bio Inc., Shiga, Japan). The first-strand cDNAs were synthesized using a ReverTra Ace qPCR RT Master Mix (Toyobo, Osaka, Japan) with 0.5 µg of total RNA. Quantitative PCR analysis was performed using a THUNDERBIRD SYBR qPCR Mix (Toyobo) followed by analysis with ABI PRISM7700 Sequence Detection System (Applied Biosystems, Foster City, CA, USA). PCR primers used in this study are listed in Table S3. To adjust the expression level of each mRNA, that of glyceraldehyde 3-phosphate dehydrogenase (GAPDH) mRNA was used as an internal control. The expression levels of GAPDH mRNA were not affected by the cisplatin treatment at the dose used in this study.

### Cell culture and gene knock-down

HEK293 cells were cultured in D-MEM containing 10% fetal bovine serum (FBS). Endoribonuclease-prepared siRNA (esiRNA) targeted to human *Asc-1* or siRNA to *ASCT2* (Sigma Aldrich, St Louis, MO, USA) was introduced to HEK293 cells using a Lipofectamine 2000 reagent (ThermoFisher scientific, Waltham, MA, USA). After incubation for 48 hours, HEK293 cells were processed for RNA extraction.

### Database search for Transcription factor binding site

DNA sequences of promoter regions (mus musculus| chr7| 35970437-35971637) for mouse *Asc-1* (gene accession, NM_017394), which include 1000 base pairs of upstream and 200 base pairs of downstream from transcription starting site (TSS) identified using DBTSS version 8.0 (https://dbtss.hgc.jp), were collected from a mouse genome database: UCSC 9 mm^54^ and uploaded to JASPAR database (http://jaspar.genereg.net). The transcription-binding sites (TFBS) were screened in JASPAR core (Table S1). Each TFBS was scored by computational prediction dependent on relevancy^55^, and cut-off value of relative score was set up higher than 0.8.

### d- and l-Serine transport assay

HEK293 cells, which were transfected with pFLAG-Asc-1, siRNA or esiRNA, or pre-treated with 1–10 µM cisplatin or 10–100 µM H_2_O_2_ for 24 h, were cultured in 10% FBS D-MEM, which contains 0.4 mM L-serine. Then, at 24 h after the transfection or pretreatment, cultured media were replaced with E-MEM, which does not contain d- or l-serine. After incubation in E-MEM for 3 hours, which totally washes out intracellular l-serine (Supplementary Fig. S5e), racemic mixtures of d- or l-serine at 100–500 µM (final conc.) were added to the media and incubated for 15–30 min. The cells were rinsed in PBS and lysed in a lysis buffer [150 mM NaCl, 50 mM Tris-HCl (pH 8.0), 1 mM EDTA, 1% TritonX-100]. Protein concentration of each sample was analyzed with BCA assay kit (ThermoFisher scientific, Waltham, MA, USA). The cell lysate was mixed with methanol at 3:7 ratio (v/v) and centrifuged at 12,100 × g for 5 min. Twenty microliter of supernatant was evaporated to dryness and resuspended in water to be processed for d- and l-serine quantification with the 2D-HPLC.

### Statistical analysis

Prism (GraphPad software) was used for data plotting and statistical analysis. Statistical significance was determined as *P* < 0.05 by Student t-test and t-test with Welch’s correction, or Pearson’s correlation coefficient (Figs 2e–g and 3 *right*). No statistical methods were used to predetermine sample size for animal experiments.

## Supplementary information

Supplementary Information
