# Supplementary material for: Serum d-serine accumulation after proximal renal tubular damage involves neutral amino acid transporter Asc-1
Source: Sci Rep. 2019 Nov 13;9:16705. doi: 10.1038/s41598-019-53302-2 (PMC6853873; doi:10.1038/s41598-019-53302-2)
Supplement: Supplementary file 1 — Supplementary Information [file 41598_2019_53302_MOESM1_ESM.pdf]

Supplemental information

Serum D-serine accumulation after proximal renal tubular damage involves neutral amino acid transporter Asc-1

Masataka Suzuki, Yusuke Gonda, Marina Yamada, Arno A. Vandebroek, Masashi Mita, Kenji Hamase, Masato Yasui, Jumpei Sasabe

Contents:

Table S1-3

Figure S1-S6

Table S1. **Informatics analysis of transcription factor binding site using JASPAR**

NM_017394, Mouse Asc-1

>M. musculus|chr7|35970437-35971637|Plus|Upstream:1000, Downstream:200

CGTGCCAGCCTCGCTCCGCCCGCCGCCCCGGTGGCCCCGACGCGGCCCTGCTC
CCTCGCACCACCCGCGCATGAATTTGCCCCGCGATCTGGGCTGCACCGCCCAAT
GGCTTCCGGGAGGAGGCAGGCTTCCCAGGATCCTGGGCCCGATACCTCCTTGCT
ACCACAATTGTTCTCTTCCCGTCTCTCTGGCACTTCCAGCGGTCTGGTCACCTTGA
GCCATGCTAGACGGCCTGGGCCAAAGTCCCAGTCCACTGTGCCCTGACTGACCT
GTGTAGAGGAGTCTGCGTAGGGGATGCTGAGGCAGGGATAGATTTGATGGTGATG
GGGACTGTCCCAAACTCCCAGGATGCCCGGGGCCCCACTGGGGTCTCCTTATCTT
CAACTCTTCTCAAGCCTTTTGCCTTCAGAATTGTTTTAAGGACAGTCTCCATTCATC
CCAAATGACTCAGTCAGGACCGCCTAGAGGCCAGGGAGCTATCAAAAAGTCTTCA
TGGAGACCGTGCCACTGTCAGGCTAATTGCCGCTAGGCGGTGGTTTTCAACCTGT
GCAATGCCCACCCTTCACAAATGGGGCTTGAGGGATTTAGAAAGTACAGGCTGGG
GCATGCAGCACCGCCTACCCCAGCACGTCTTGCTCACACTCCTTGAGCGCAGGG
ACAGCCTGGTCTTTGAGCTCTTCTCCCCCACCTCACCCAAGTCTGGGTGTAGCCC
CTTCGTTCACTCAACTAGGAAGTCCTTTCTAAGTCTGATCCCAGTCCCCTCCTGTC
TCCCCTGACTAGGGTTGGGGGTGGGGGGGAAGGGGGAGTTCCCTTTAAGGGCG
GTGGGGGAGGGGGCTGCCGGCCGTCCCTCCTTCCGTCCCTCCCTGGATCCAGC
CGCCCGGGGGCCCCCGGGGGCCCGGGCCCCGTGCCAGCGCATGCGCCCGCCT
GTGGGCGCTGTCCCGGGCTGCGAGGGCCGCGAGCGCACCGACAGACGAACCGA
CCGACGGAGGACTGGCTGCGGGCCGGACAGACGGGGTAGCGCAGGGAACTGG
GATGAGGCGGGACAGCGACATGGCAAGCCACATACAACAGCCAGGCGGGCACGG
GAACCCCGGCCCTGCGCCCTCGCCTTCCCCGGGCCCTGGTCCCGGCCCGGGCG
CCTCGGAGCGGGTGGCACTCAAGAAAGAGAT

Scores more than 10 are highlighted (blue, green, and red).

Yellow: Transcription start site

Blue: STAT3 binding site

Green: NF-kB binding site

Red: JUN::FOS binding site

Table S2. **Primer pairs used in cloning, sub-cloning and sequence confirmation**

Target gene	Accession number	PCR	5’ Forward	3’ Reverse
ASCT2	NM_018861.3	cloning	ATGGCAGTGGATCCCCCTAA	TCACATGACAGATTCCTTTTCGAA
		Infusion sub-cloning	ATCAGTCGACGGATCCACCATGGATCCCCCTAA	AATCGGTACCGGATCCCATGACAGATTCCTTTTCGAA
Asc-1	NM_017394.4	cloning	ATGAGGCGGGACAGCGAC	TCATTGTGTCTTCAAGGGCTTG
		Infusion sub-cloning	ATCAGTCGACGGATCCACCATGAGGCGGGACAGCGAC	AATCGGTACCGGATCCTTGTGTCTTCAAGGGTTG

Primers for sequence confirmation

Target gene	Primer name	sequence
ASCT2	(N-CMV30)	AATGTCGTAATAACCCCGCCCCGTTGACGC
ASCT2	(C-CMV24)	TATTAGGACAAGGCTGGTGGGCAC
Asc-1	(N-CMV30)	AATGTCGTAATAACCCCGCCCCGTTGACGC
Asc-1	Asc-1 450-469 seq	CTATGTGCTTCAGCCTGTCT
Asc-1	Asc-1 1001-1020 seq	GAGGGATCAATGGCTACCTG

Table S3. **Primer pairs used in qPCR**

Target gene	Organisms	Accession number	5' Forward	3' Reverse
ASCT1	mouse	NM_018861.3	GGCATCGCTGTTGCTTACTTC	CGAGGAAAGAGTCCACTGTCT
ASCT2	mouse	NM_009201.2	CATCAACGACTCTGTTGTAGACC	CGCTGGATACAGGATTGCGG
4F2hc	mouse	NM_008577.4	TGATGAATGCACCCTTGTACTTG	GCTCCCCAGTGAAAGTGGA
ATB(0+)	mouse	NM_020049.4	GACAGCTTCATCCGAGAACTTC	ATTGCCCAATCCCACTGCAT
B^0AT1	mouse	NM_028878.3	CAGGTGCTCAGGTCTTCTACT	CGATCACAGAATCCATCTCACAA
B^0AT3	mouse	NM_001040692.3	TGCTTTGCCTGTTTCCTCTCA	ATGTCATCACAGAACCGTTTCAT
LAT2	mouse	NM_016972.2	TGTGACTGAGGAACTTGTGGA	GTGGACAGGGCAACAGAAATG
Asc-1	mouse	NM_017394.4	GGGTTTGGCCCTCTTCGTC	GACATAGGCGTAGTCCCCAC
TAT1	mouse	NM_001114332.1	GAGGTGGAGCTGACGAGGT	GTGAAGACACTCACGATGGGG
SNAT2	mouse	NM_175121.3	TAATCTGAGCAATGCGATTGTGG	AGATGGACGGAGTATAGCGAAAA
SNAT4	mouse	NM_027052.3	GCGGGGACAGTATTCAGGAC	GGAACTTCTGACTTTCGGCAT
SNAT5	mouse	NM_172479.3	ACCTGCCGGGAAAGTAGTC	AGGAAGGTGCCAATAACAAGG
GAPDH	mouse	NM_008084.3	ACCACAGTCCATGCCATCAG	TCCACCACCCTGTTGCTGTA
ASCT2	human	NM_005628.2	CCGCCTTGGCAAGTACATTCT	GGCAGGATGAAACGGCTGA

Asc-1	human	NM_019849.2	GCCTGCACCATCATCATCG	GTGACGTAGGCGTAGTCCC
GAPDH	human	NM_002046.6	ATGGGGAAGGTGAAGGTCG	GGGGTCATTGATGGCAACAATA

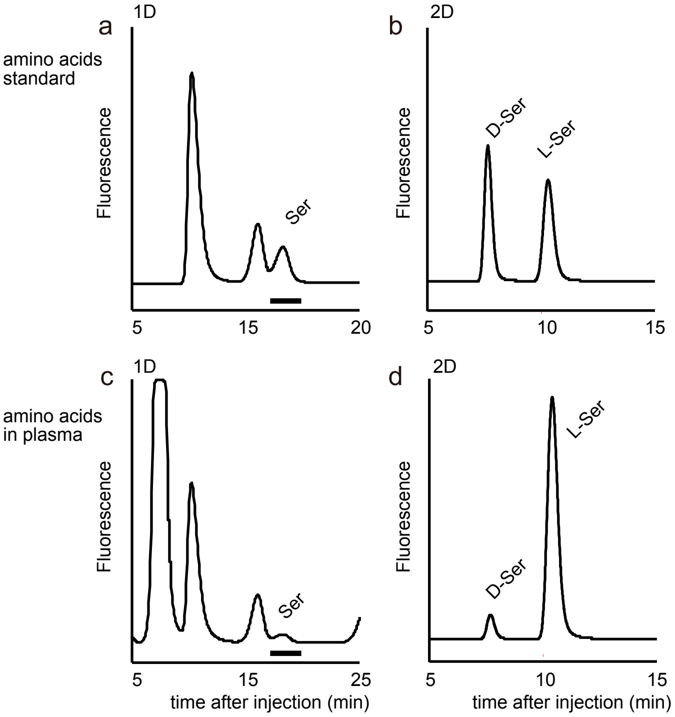

Figure S1. Representative chromatograms of serine enantiomers in 2D-HPLC. **a-d**, D-/L-serine standards (a, b) or plasma D-/L-serine (c, d) were analyzed in the first dimensional separation (a, c) and in the second dimensional separation (b, d). Black bars in the first dimensional chromatogram indicate the time window when samples were injected into the second dimensional column. 'Ser' indicates D- and L-serine.

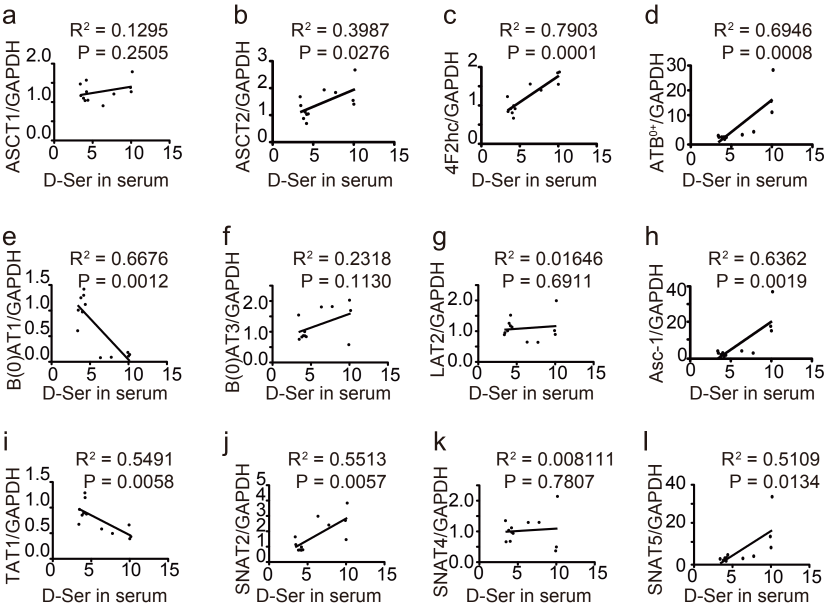

Figure S2. Correlations of renal transcriptions of neutral amino acid transporters with serum D-serine levels in cisplatin-treated animals. Pearson's correlations between concentration of D-serine (μM) in the serum and relative amount of mRNA expressions of ASCT1 (**a**), ASCT2 (**b**), 4F2hc (**c**), ATB^{0+} (**d**), B^{0}AT1 (**e**), B^{0}AT3 (**f**), LAT2 (**g**), Asc-1 (**h**), TAT1 (**i**), SNAT2 (**j**), SNAT4 (**k**), and SNAT5 (**l**) in the kidney of cisplatin-treated animals are shown.

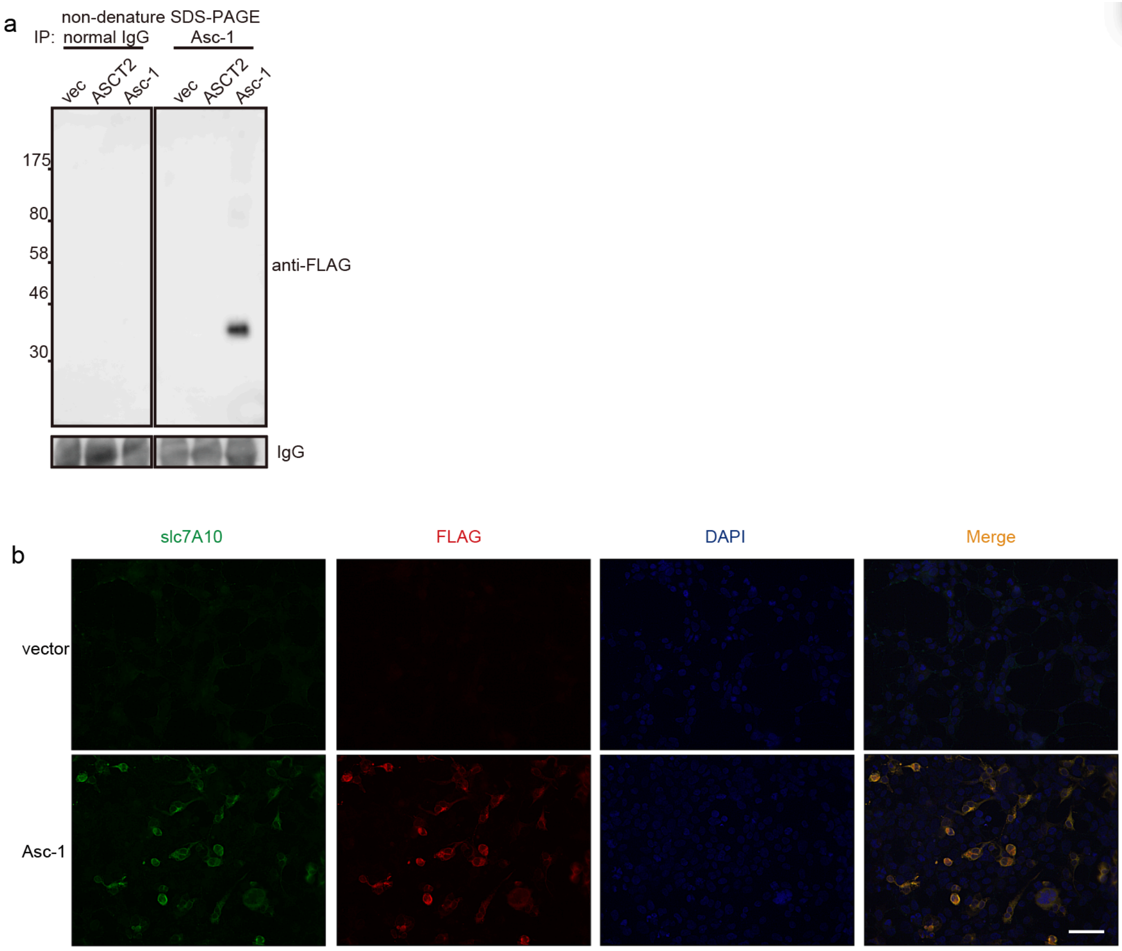

Figure S3. Characterization of an antibody raised against mouse Asc-1. **a**, Cell lysate from HEK293 cells overexpressed with FLAG-tagged Asc-1, ASCT2, or vector control were immunoprecipitated with rabbit normal IgG (left) or a rabbit antibody to Asc-1 (right) and detected with a mouse monoclonal antibody to FLAG. **b**, HEK293 cells overexpressed with FLAG-tagged Asc-1 or control were labeled with the rabbit polyclonal antibody to Asc-1 (green), the mouse monoclonal antibody to FLAG (red), and DAPI (blue). Scale bar, 50 μm.

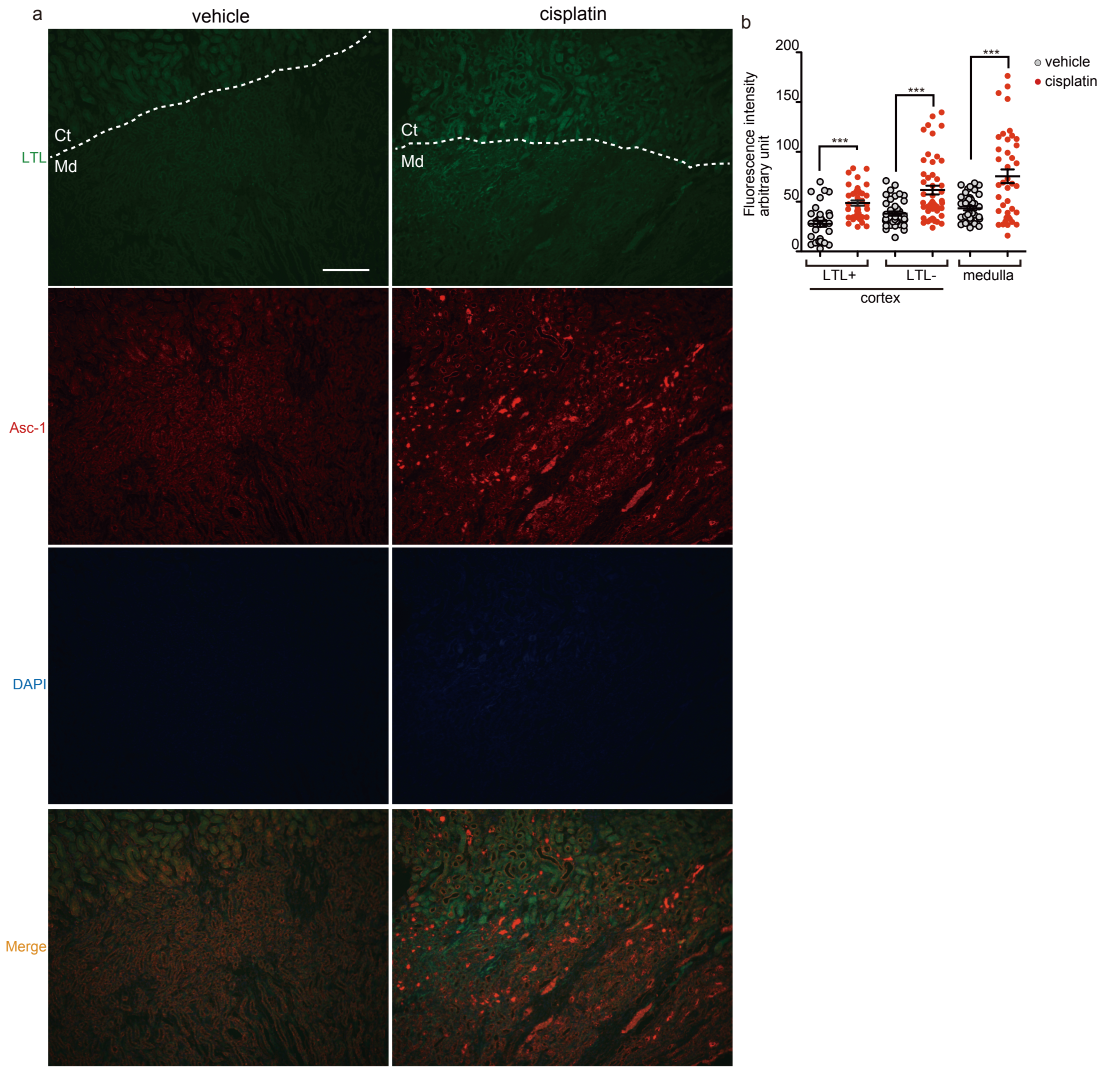

Figure S4. Histological distribution of Asc-1 in kidney slices from mice with severe renal dysfunction. **a**, LTL (green), Asc-1 (red), and DAPI (blue) were stained using kidney slices from vehicle- and cisplatin-treated mice. Right panels show merged images. White dotted lines are borders between renal cortex (Ct) and medulla (Md). Scale bar, 200 μm. **b**, The fluorescence intensity of Asc-1-labelling in the nephron (LTL-positive or LTL-negative tubules in the cortex, or medulla) were quantified (36-50 renal tubules in each 3 mice per each group). Error bars, mean ± s.e.m. Mann-Whitney U test, ***$P < 0.001$.

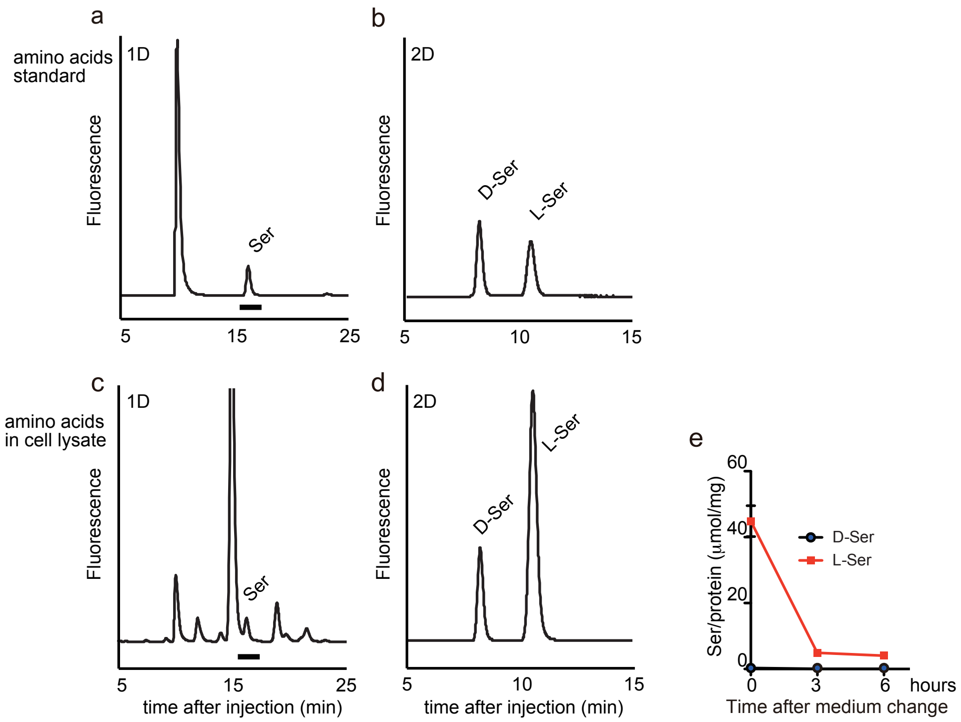

Figure S5. Representative chromatograms of serine enantiomers in the cell culture in the 2D-HPLC. **a-d**, D-/L-serine standards (a, b) or intracellular D-/L-serine (c, d) were analyzed in the first dimensional separation (a, c) and in the second dimensional separation (b, d). Black bars in the first dimensional chromatogram indicate the time window when samples were injected into the second dimensional column. 'Ser' indicates D- and L-serine. **e**, Intracellular serine enantiomers in HEK293 cells were monitored using 2D-HPLC after replacement of D-MEM medium containing 10% fetal bovine serum with serum-free E-MEM.

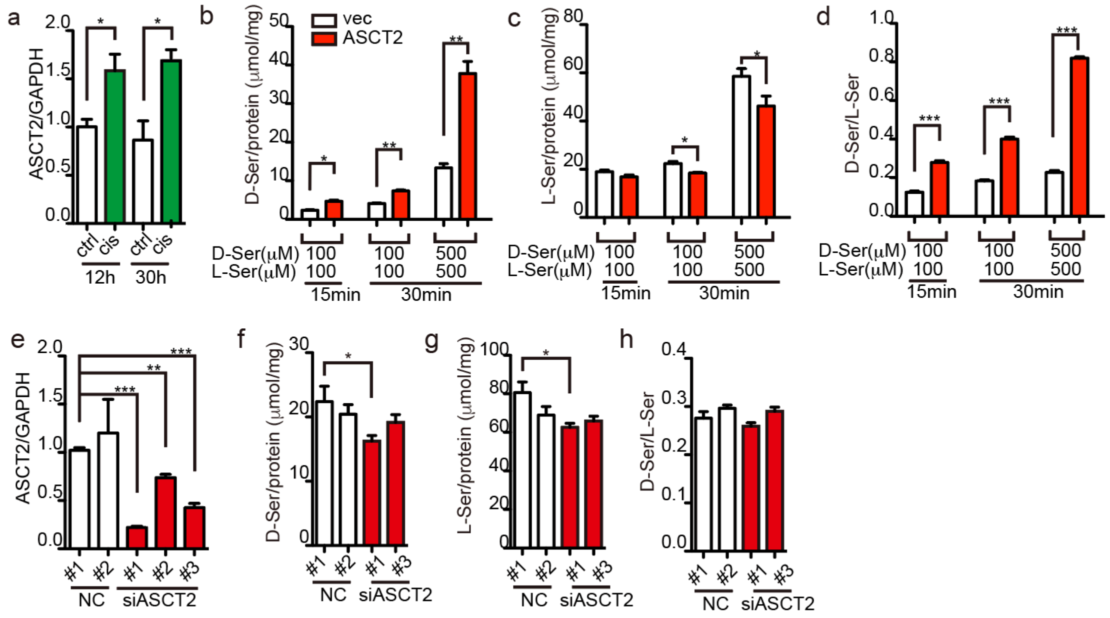

Figure S6. Involvement of ASCT2 in D-serine transport under cisplatin treatment. **a**, Expression of mRNA for ASCT2 after treatment with 10 μM cisplatin or H_2O_2 for 15 h was evaluated with qPCR and standardized with mRNA levels of GAPDH. **b-d** and **f-h**, Inward transport of D-/L-serine in the HEK293 cells was measured using 2D-HPLC after overexpression (b-d) or knockdown of ASCT2 (f-h). **e**, Expression of mRNA for ASCT2 at 24 h after transfection with siRNAs for ASCT2 or their controls was evaluated with qPCR and standardized with mRNA levels of GAPDH. 'cis', cisplatin. 'ctrl', control. n = 4. Error bars, mean± s.e.m. Student's t-test. $^*P < 0.05$, $^{**}P < 0.01$, $^{***}P < 0.001$.
